# Related Factors and Clinical Outcomes of Osteosarcopenia: A Narrative Review

**DOI:** 10.3390/nu13020291

**Published:** 2021-01-20

**Authors:** Tatsuro Inoue, Keisuke Maeda, Ayano Nagano, Akio Shimizu, Junko Ueshima, Kenta Murotani, Keisuke Sato, Kazuki Hotta, Shinichiro Morishita, Atsuhiro Tsubaki

**Affiliations:** 1 Department of Physical Therapy, Niigata University of Health and Welfare, 1398 Shimami-cho, Kita-ku, Niigata 950-3198, Japan; tatsuro-inoue@nuhw.ac.jp (T.I.); kazuki-hotta@nuhw.ac.jp (K.H.); morishita@nuhw.ac.jp (S.M.); tsubaki@nuhw.ac.jp (A.T.); 2 Department of Geriatric Medicine, National Center for Geriatrics and Gerontology, 7-430 Morioka, Obu, Aichi 474-8511, Japan; 3 Department of Palliative and Supportive Medicine, Graduate School of Medicine, Aichi Medical University, 1-1 Yazakokarimata, Nagakute, Aichi 480-1195, Japan; 4 Department of Nursing, Nishinomiya Kyoritsu Neurosurgical Hospital, 11-1 Imazuyamanaka-cho, Nishinomiya, Hyogo 663-8211, Japan; aya.k.nagano@gmail.com; 5 Department of Nutrition, Hamamatsu City Rehabilitation Hospital, 1-6-1 Wago-kita, Naka-ku, Hamamatsu, Shizuoka 433-8127, Japan; a.shimizu.diet@gmail.com; 6 Department of Clinical Nutrition and Food Service, NTT Medical Center Tokyo, 5-9-22 Higashi-Gotanda, Shinagawa-ku, Tokyo 141-8625, Japan; j.ueshima@gmail.com; 7 Biostatistics Center, Kurume University, 67 Asahimachi, Kurume 830-0011, Japan; kmurotani@med.kurume-u.ac.jp; 8 Okinawa Chuzan Hospital Clinical Research Center, Chuzan Hospital, 6-2-1 Matsumoto, Okinawa 904-2151, Japan; keisuke.sato0815@gmail.com

**Keywords:** sarcopenia, osteoporosis, osteosarcopenia

## Abstract

Osteopenia/osteoporosis and sarcopenia are common geriatric diseases among older adults and harm activities of daily living (ADL) and quality of life (QOL). Osteosarcopenia is a unique syndrome that is a concomitant of both osteopenia/osteoporosis and sarcopenia. This review aimed to summarize the related factors and clinical outcomes of osteosarcopenia to facilitate understanding, evaluation, prevention, treatment, and further research on osteosarcopenia. We searched the literature to include meta-analyses, reviews, and clinical trials. The prevalence of osteosarcopenia among community-dwelling older adults is significantly higher in female (up to 64.3%) compared to male (8–11%). Osteosarcopenia is a risk factor for death, fractures, and falls based on longitudinal studies. However, the associations between osteosarcopenia and many other factors have been derived based on cross-sectional studies, so the causal relationship is not clear. Few studies of osteosarcopenia in hospitals have been conducted. Osteosarcopenia is a new concept and has not yet been fully researched its relationship to clinical outcomes. Longitudinal studies and high-quality interventional studies are warranted in the future.

## 1. Introduction

As the aging population grows, the number of older adults with multiple comorbidities has increased. Age-related deterioration of the structure and function of the musculoskeletal system, which is composed of bone and muscle, is common in the elderly [1,2]. The musculoskeletal system plays an important role not only in activities of daily living (ADL), such as walking, but also in metabolism [3]. Reduced functioning of the musculoskeletal system leads to falls and fractures [4] and significantly reduces ADL and quality of life (QOL) [5].

Osteopenia/osteoporosis and sarcopenia are common geriatric diseases among older adults that harm ADL and QOL. Osteopenia/osteoporosis is a condition of reduced bone mineral density (BMD) [3] that is strongly associated with the development of fragility fractures [4]. Meanwhile, sarcopenia is a skeletal muscle disease that presents with muscle mass loss, muscle weakness, and loss of physical function [6]. Sarcopenia is strongly associated with falls, disability, disease, hospitalization, and death [7]. Osteopenia/osteoporosis and sarcopenia have proven their association in a meta-analysis [8]. Therefore, considering both osteopenia/osteoporosis and sarcopenia at the same time is a key strategy to prevent disability and poor QOL. In order to link osteopenia/osteoporosis with sarcopenia, a new concept called osteosarcopenia has recently emerged.

Osteosarcopenia is a unique syndrome that is a concomitant of both osteopenia/osteoporosis and sarcopenia [9]. Bones and muscles show strong interactions with each other [10]. Osteopenia/osteoporosis and sarcopenia have common risk factors, such as aging; sex; inactivity; reduced vitamin D; the growth hormone, insulin-like growth factor I; and testosterone [10]. However, given osteosarcopenia is a new concept, its epidemiology has not yet been fully researched.

Understanding the relationship between osteosarcopenia and clinical outcomes is essential for the development of clinical research on the topic, but this relationship is not yet fully understood. Most reviews have focused on the common pathological factors of osteopenia/osteoporosis and sarcopenia and have explained the pathogenesis of osteosarcopenia. This review aimed to summarize the related factors and clinical outcomes of osteosarcopenia to facilitate the understanding, evaluation, prevention, treatment, and further research on osteosarcopenia.

## 2. Materials and Methods

### 2.1. Data Sources and Search Strategy

This review was conducted in adherence to the guidelines of the Preferred Reporting Items for Systematic Reviews and Meta-Analyses (PRISMA) [11]. We conducted a literature search using PubMed (MEDLINE), Cochrane Central Register of Controlled Trials (CENTRAL). The following search terms were used for the literature search: Sarcopenia, osteopenia, osteoporosis, and osteosarcopenia.

### 2.2. Study Selection

#### 2.2.1. Inclusion Criteria

We set the following inclusion criteria for the included studies in this review: (1) Studies including participants in any setting, including those living in communities and hospitalized; (2) studies including participants of all sexes and races; (3) studies examining the impact of osteosarcopenia on clinical outcomes; (4) studies comprising clinical outcomes including death, fracture, falling, frailty, chronic diseases, physical functions, nutritional status, endocrine system, mobility, and any other clinical outcome; and (5) observational and intervention studies.

#### 2.2.2. Exclusion Criteria

Editorials, case reports, letters to the editor, animal studies, and conference abstracts were excluded.

### 2.3. Data Extraction

We extracted the following information from the included studies: the names of the first author; year of publication; country of origin; study design; study setting; study sample size; age of participants; sex prevalence; measurement of muscle mass, muscle strength, and BMD; diagnosis criteria including sarcopenia, osteoporosis/osteopenia, and osteosarcopenia; osteosarcopenia prevalence; main study outcomes; and main results.

### 2.4. Quality Assessment

We assessed the quality of the included study using both the National Institutes of Health Quality Assessment tool for Observational Cohort and Cross-Sectional Studies and the Quality Assessment of Controlled Intervention Studies [12]. This quality assessment tool consisted of 14 assessment items in each study design. We have scored these items and classified the included studies as “good,” “fair,” or “poor.” (Supplementary Table S1 and S2).

## 3. Results

### 3.1. Diagnosis

#### 3.1.1. Osteopenia/Osteoporosis

Osteopenia/osteoporosis is diagnosed using the World Health Organization (WHO) criteria [13], and bone mineral density (BMD) is measured by dual X-ray absorptiometry (DXA). Osteopenia is defined as a BMD between −1.0 and −2.5 in standard deviations (SDs) for a young healthy adult (T-score), and osteoporosis as a BMD of −2.5 SDs or below [13]. Some studies on Japanese individuals have used the Japan Osteoporosis Society (JOS) criteria. Osteoporosis by JOS criteria is defined as a mean BMD of young adults <70% [14]. Even when the JOS criterion is used, there is little difference from the values of a T-score less than −2.5 SD as presented in the WHO criteria [14]. 

#### 3.1.2. Sarcopenia

Sarcopenia has been defined as the presence of low muscle mass and low muscle strength and/or physical function [7]. The most widely used diagnostic criteria are the European Working Group on Sarcopenia in Older People (EWGSOP) criteria [15], updated EWGSOP criteria [16], the Asian Working Group for Sarcopenia (AWGS) criteria [17], and the updated AWGS 2019 [18]. DXA is recommended as the preferred instrument for muscle mass measurement in each criterion. When using DXA, the cut-off value for muscle mass in the updated EWGSOP criteria is 7.0 kg/m^2^ for males and 5.5 kg/m^2^ for females [16], and the AWGS 2019 criteria is 7.0 kg/m^2^ for males and 5.4 kg/m^2^ for females [18]. Muscle strength is measured by grip strength. Gait speed, the 5-time chair stand test, and the Short Physical Performance Battery (SPPB) are recommended to measure physical function [16,18].

#### 3.1.3. Osteosarcopenia

Osteosarcopenia is a syndrome in which osteopenia/osteoporosis and sarcopenia coexist [9]. The criteria are not consistent, given some studies refer to osteopenia and sarcopenia, whereas others refer to osteoporosis and sarcopenia as osteosarcopenia.

Osteosarcopenic obesity is a syndrome in which osteosarcopenia and obesity coexist [19]. Definition of obesity is based on the WHO criteria of >35% body fat for women younger than 60 years [20] and >40% body fat for women aged 60 years and older [21]; for osteosarcopenic obesity [22].

### 3.2. Prevalence of Osteosarcopenia

The prevalence of osteosarcopenia among community-dwelling older adults is significantly higher in women compared with men. It has been reported that up to 64.3% of women had osteosarcopenia, compared with 8–11% of men among community-dwelling older adults (Table 1). Women have reduced estrogen secretion after menopause and are more likely to suffer from osteoporosis [4].

Few studies of osteosarcopenia in hospitals have been conducted. Studies have been conducted only on hip fractures, liver disease, and primary biliary cholangitis (Table 2). In female hip fracture patients, the prevalence of osteosarcopenia was 28.7–65.7% [35,36]. The prevalence of osteosarcopenia in patients with liver disease was 16.8–21.8% [37,38] and 15.4% in patients with primary biliary cholangitis [39].

### 3.3. Osteosarcopenia as a Risk of Poor Clinical Outcomes

**Figure 1:**
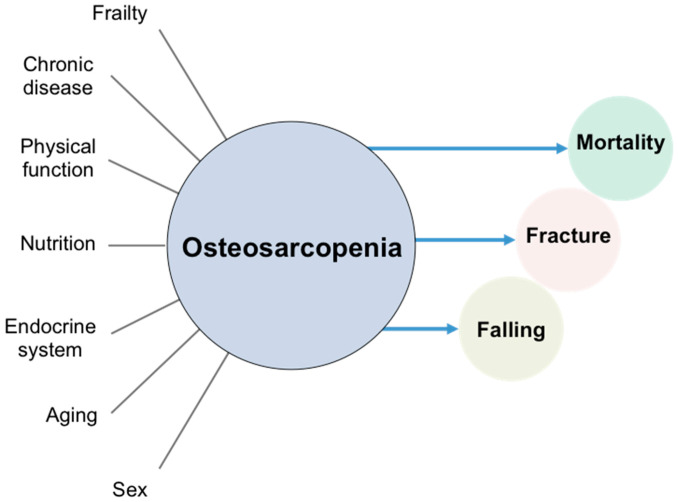
Osteosarcopenia and related factors. Lines indicate cross-sectional associations and arrows indicate longitudinal associations.

Osteosarcopenia is associated with many factors. Osteosarcopenia is a risk factor for death, fractures, and falls based on longitudinal studies. However, given the associations between osteosarcopenia and many other factors have been derived based on cross-sectional studies, the causal relationship is not yet clear (Figure 1).

#### 3.3.1. Mortality

Osteosarcopenia is a risk factor for mortality. In a prospective observational study of 1032 community-dwelling older adults in Australia, osteosarcopenia had significantly higher 10-year mortality compared with those without sarcopenia or osteopenia (relative risk [RR] 1.49, 95% CI 1.01−2.21) [30]. Also, in a study of community-dwelling older adults in Chile, osteosarcopenia was associated with significantly higher mortality compared with those without sarcopenia or osteopenia (hazard ratio [HR] 1.81, 95% CI 1.09−2.98) [34]. In a study of hip fractures, those with osteosarcopenia had a higher risk of mortality than those with sarcopenia, osteoporosis, and normal BMD [35]. These results suggest that osteosarcopenia is a risk factor for mortality in community-dwelling older adults and patients with hip fractures.

#### 3.3.2. Falls and Fractures

**Figure 2:**
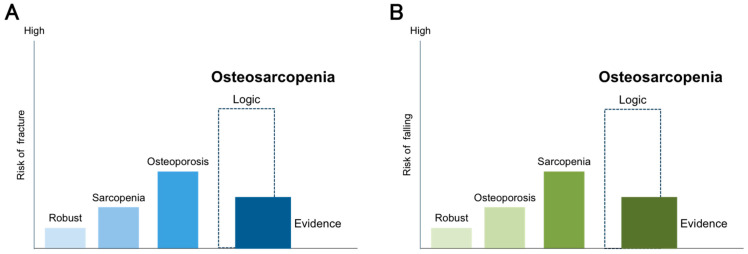
Gaps between logic and evidence as predictors of fractures and falls in osteosarcopenia. Logic dictates that osteosarcopenia, which is a combination of sarcopenia and osteoporosis, may have a synergistic effect on fractures and falls. On the other hand, evidence from previous studies shows that osteosarcopenia had only a small or no effect on fractures and falls. (**A**) Risk of fracture, (**B**) Risk of falling.

An association between osteosarcopenia and falls or fractures has been reported from longitudinal [28,30,34] and cross-sectional [24,33] studies. However, we must interpret osteosarcopenia carefully. Scott D et al. reported that osteosarcopenia was a risk factor for falls for 2 years and fractures for 6 years compared with those without sarcopenia and osteopenia in community-dwelling older men [28]. However, in this study, the HR for falls was higher for sarcopenia alone (HR 1.61; 95% CI 1.14−2.28) than for osteosarcopenia (HR 1.41; 95% CI 1.02−1.95). Interestingly, osteosarcopenia was not a risk factor for hip fractures (HR 1.84; 95% CI 0.60−5.61); only osteoporosis alone was a risk factor for hip fractures (HR 2.58; 95% CI 1.22−5.45). In another prospective observational study of community-dwelling older adults, osteoporosis alone was associated with fractures over a period of 10 years (RR 1.50; 95% CI 1.07−2.10), but osteosarcopenia was not (RR 1.48; 95% CI 0.83−2.64) [30]. Thus, osteosarcopenia might not show a synergistic effect of osteoporosis and sarcopenia for predicting falls and fractures (Figure 2). The reasons for the discrepancy between the logic and evidence are not clear. Prospective, well-designed observational studies are warranted.

#### 3.3.3. Frailty

The association between frailty and osteosarcopenia has been well investigated; however, the association appears to vary depending on the definition of frailty. The frailty phenotype [41] is frequently used to define frailty and has been reported to be associated with osteosarcopenia [8,23]. An association between frailty and osteosarcopenic obesity has also been reported. Szlejf C et al. reported that frailty defined by the frailty phenotype and the Gerontopole Frailty Screening Tool were associated with osteosarcopenic obesity, but not the Fatigue, Resistance, Ambulation, Illness, and Loss of weight scale [22]. The frailty phenotype is frequently used for frailty diagnosis, and it might be a suitable diagnostic criterion for considering osteosarcopenia and osteosarcopenic obesity.

#### 3.3.4. Comorbidity

An association between osteosarcopenia and chronic diseases has been reported. The associations between osteosarcopenia and peptic disease [24], inflammatory arthritis [24], diabetes (only in men) [23], and kidney dysfunction [8] have been reported. There are reports of an association between a number of chronic diseases (3 or more) [23] and osteosarcopenia. There has also been a report that high levels of HbA1c are a risk factor for osteosarcopenia [8]. Regarding the psychological aspect, depression as assessed using the Geriatric Depression Scale was associated with osteosarcopenia [24]. Osteoporosis and sarcopenia have been associated with inflammatory cytokines [10] and dietary inflammatory index scores [27], a measure of the impact of diet on inflammatory status. Hence, it is likely that chronic diseases have an impact on the development and severity of osteosarcopenia.

#### 3.3.5. Nutritional Status

Most studies have used body mass index (BMI) as an indicator of nutritional status. Okamura H et al. and Fahimfar N et al. reported that the risk of osteosarcopenia increases with decreasing BMI [8,32], but they did not report a clear cut-off value. Reiss J et al. reported significantly lower Mini Nutritional Assessment-Short Form scores among hospitalized elderly patients with osteosarcopenia compared with sarcopenia alone and osteoporosis alone [40]. To validate the accurate association between nutritional status and osteosarcopenia, it is necessary to use validated nutritional screening tools [42] and Global Leadership Initiative on Malnutrition criteria [43], in order to assess the risk and to diagnose undernutrition.

#### 3.3.6. Physical Function

Osteosarcopenia has a negative impact on physical function. Osteosarcopenia is associated with grip strength [31], chair rising time [25], sit to stand power [25], the SPPB [33], the Timed Up and Go test [33], and the Four-Square Step test [33]. In addition, Salech F et al. reported that osteosarcopenia is a risk factor for functional limitation in community-dwelling older adults [34]. Osteosarcopenia was also associated with poorer chair rising time and sit-to-stand power, but not with sarcopenia alone or osteoporosis alone [25]. It has also been reported that osteosarcopenic obesity was associated with SPPB [22]. Given physical function is included in the diagnostic criteria for sarcopenia, a strong association between osteosarcopenia and physical function is to be expected.

#### 3.3.7. Endocrine System and Bone Metabolism Marker

Endocrine factors are important physiological and pathological factors for maintaining bone and muscle structure and function [10]. In cross-sectional studies, an association between osteosarcopenia and serum parathyroid hormone [26] and insulin-like growth factor 1 [29] has been reported. In addition, an association between osteocalcin, b-crosslaps, and procollagen type 1 amino-terminal propeptide [25] and osteosarcopenia has been reported as a bone metabolism marker.

### 3.4. Intervention

Several randomized controlled trials (RCTs) have been conducted on community-dwelling older adults with osteosarcopenia. The first RCT examined the effects of 28 weeks of continuous high-intensity resistance training in community-dwelling osteosarcopenic men aged ≥72 years [44]. The results showed a significant positive effect on the sarcopenia Z-score (*p* < 0.001) in the intervention group and a significant worsening in the control group (*p* = 0.012). There was a significant increase in the skeletal muscle index (SMI) only in the intervention group and a significant intergroup difference in SMI and handgrip strength (both *p* < 0.001). The second RCT was also conducted in community-dwelling osteosarcopenic men, who received high-intensity dynamic resistance training (HIT-DRT) and whey protein supplementation for 18 months; the effects on BMD and sarcopenia parameters were examined [45]. The results showed that the effect sizes for skeletal muscle mass changes were pronounced (1.97, *p* < 0.001), whereas the effects for functional sarcopenia parameters were moderate (0.87, *p* = 0.008; handgrip strength) or low (0.39, *p* = 0.209; gait velocity). A third study similarly examined the impact of 12 months of low-volume HIT-DRT on BMD and SMI in men with osteosarcopenia [46]. The results showed that lumbar spine BMD was maintained in the intervention group but decreased in the control group, with a difference between groups (*p* < 0.001). SMI increased significantly in the intervention group but decreased significantly in the control group (both *p* < 0.001). Hence, resistance training alone or in combination with resistance training and protein were effective against osteoporosis or sarcopenia for community-dwelling osteosarcopenic men. However, there are no RCTs of osteosarcopenic women at this time. Because osteosarcopenia is more common in women, RCTs of osteosarcopenic women are desirable.

## 4. Call for Action

Osteosarcopenia is a new concept, and few studies have been performed on its association with clinical outcomes. In this review, we summarize the association between osteosarcopenia and clinical outcomes to facilitate its understanding, evaluation, prevention and treatment. As a result, we found several issues for clinical and research advancement in osteosarcopenia. First, we identified most of the factors of osteosarcopenia restricted cross-sectional associations. Longitudinal studies for the future occurrences of osteosarcopenia and the impact of osteosarcopenia on clinical outcomes in community-dwelling older adults should lead to efficient prevention and treatment. In recent years, DXA has been widely used to assess muscle mass in community-dwelling older adults. Focusing on BMD, as well as muscle mass, can lead to research on osteosarcopenia. Second, the associations between osteosarcopenia and cognitive functioning, psychological aspects, and social skills have not yet been well examined. Given these factors have been specifically reported to be associated with sarcopenia, they could also be associated with osteosarcopenia. Third, high-quality intervention studies are needed. For women in particular, certain drugs that have been shown to be effective in treating osteoporosis could further improve clinical outcomes. Fourth, we need to aggressively conduct clinical studies in a hospital setting, given very few of these have been performed. At minimum, more studies on osteoporotic fractures (e.g., hip fractures, vertebral fracture) are warranted. Fifth, as an important step to resolve these issues, the development of a simple tool to screen for osteosarcopenia is necessary. The development of a simple screening tool can help clinical and research progress in osteosarcopenia. Finally, a meta-analysis would be required to elucidate the related factors and clinical outcomes of osteosarcopenia.

## 5. Conclusions

This review summarized the related factors and clinical outcomes of osteosarcopenia to facilitate its understanding, evaluation, prevention, treatment, and further research. Osteosarcopenia was a risk factor for fractures and falls and had cross-sectional associations with age, sex, frailty, chronic disease, physical function, nutrition, and the endocrine system. Longitudinal studies and high-quality interventional studies on elderly community-dwelling adults and hospitalized patients are warranted.

## Figures and Tables

**Table 1:** Assessment, prevalence, and impact of osteosarcopenia on clinical outcomes in community-dwelling older adults.

Author, Year, Country	Design, Setting	Age Sample Size Male/Female, n (%)	Measurements	Diagnosis	Prevalence	Outcomes	Main Results
Wang, Y.J. et al. 2015 [23] China	Cross-sectional study, Community-dwelling	Men: 75.6 ± 4.8 Women: 74.9 ± 5.2 316 164(51.9)/152(41.8)	Muscle mass: BIA Muscle strength: grip strength BMD: DXA	Sarcopenia: AWGS Osteoporosis: WHO criteria	Men: 10.4% Women: 15.1%	Frailty (Frailty Phenotype)	≥80 years old (OR 4.8; 95% CI, 3.05–10.76), women (OR 2.6; 95% CI, 1.18–2.76), and higher level of comorbidity (OR 3.71; 95% CI) were independently associated with the likelihood of being osteosarcopenia. The likelihood of being frail/prefrail was substantially higher in the presence of osteosarcopenia (OR 4.16; 95% CI, 2.17–17.65 in men; and OR 4.67; 95% CI, 2.42–18.86 in women).
Huo YR et al. 2015 [24] Australia	Cross-sectional study, Patients referred to the Falls and Fractures Clinic	Mean 79 *N* = 679 224(35)/455(65)	Muscle mass: DXA Muscle strength: grip strength BMD: DXA	Sarcopenia: EWGSOP Osteopenia, osteoporosis: WHO criteria	37%	Depression Nutritional status Comorbidity History of trauma fracture Mobility	Osteosarcopenia patients are older, mostly women, are at high risk for depression and malnutrition, have BMI < 25, and showed a higher prevalence of peptic disease, inflammatory arthritis, maternal hip fracture, history of a traumatic fracture, and impaired mobility.
Drey M et al. 2016 [25] Germany	Cross-sectional study, Community-dwelling	Osteosarcopenia: 78 ± 7.5 Sarcopenia: 76 ± 6.1 Osteopenia/osteoporosis: 81 ± 5.0 Control: 74 ± 6.4 *N* = 68 Osteosarcopenia: 32%/68% Sarcopenia: 29%/71% Osteopenia/osteoporosis: 29%/71% Control: 33%/67%	Muscle mass: DXA BMD: DXA	The 50th percentile of each sex Sarcopenia: aLM Female: <6.398 kg/m^2^ Male: <7.367 kg/m^2^ Osteopenia/osteoporosis T-score Female: <−0.6, Male: <−0.9	27.9%	Physical performance Bone turnover	Only osteosarcopenia showed significantly reduced hand grip strength, increased chair rising time, and sit to stand power time as well as significantly increased bone turnover markers.
Szlejf C et al. 2017 [22] Mexico	Cross-sectional study (of a prospective cohort), Community-dwelling	Mean 71.3 ± 9.5 *N* = 427 (all women)	Muscle mass: DXA Muscle strength: grip strength BMD: DXA	Sarcopenia: FNIH criteria Osteopenia, osteoporosis: WHO criteria Obesity: WHO criteria (>35% body fat < 60 years, >40% body fat ≥ 60 years)	Osteosarcopenic obesity 19% (N = 81)	SPPB Frailty (Frailty Phenotype, GFST, the FRAIL scale)	Frailty (according to the Frailty Phenotype and the GFST) and poor physical performance measured by the SPPB were independently associated with osteosarcopenic obesity, controlled by age.
Suriyaarachchi P et al. 2018 [26] Australia	Cross-sectional study, Patients referred to the Falls and Fractures Clinic	Mean 79 *N* = 400	Muscle mass: DXA Muscle strength: grip strength BMD: DXA	Sarcopenia: EWGSOP Osteopenia, osteoporosis: WHO criteria	40%	Serum PTH	Subjects with high PTH levels were more likely to be in the osteosarcopenia than in the non-sarcopenic or non-osteopenic (OR 6.88; CI: 1.9–9.2).
Susan Park et al. 2018 [27] Korea	Cross-sectional study, Community-dwelling	Mean 62.34 ± 0.31 *N* = 1344 (all women)	Muscle mass: DXA BMD: DXA	Sarcopenia: ASM less than 1 SD below the average of women aged 20–40 years Osteopenia, osteoporosis: WHO criteria Obesity: BMI >25 kg/m^2^	Osteosarcopenia: 314 (24.1%) Osteosarcopenic obesity: 455 (31.8%)	Dietary inflammatory index scores	Women with higher dietary inflammatory index scores were more likely to have risk of osteopenic obesity (OR = 2.757, 95% CI: 1.398–5.438, *p* < 0.01) and that of osteosarcopenic obesity (OR = 2.186, 95% CI: 1.182–4.044, *p* < 0.05).
Scott D et al. 2019 [28] Australia	Observational study, Community-dwelling	Mean 76.7 ± 5.4 *N* = 1575 (all men)	Muscle mass: DXA Muscle strength: grip strength BMD: DXA	Sarcopenia: EWGSOP Osteopenia, osteoporosis: WHO criteria Obesity: BMI >25 kg/m^2^	8%	Incident fractures (6 ± 2 years) Incident falls (for 2 years)	Only men with osteosarcopenia had significantly increased fall (RR 1.41; 95% CI: 1.02 to 1.95) and fracture risk (HR: 1.87; 95% CI: 1.07 to 3.26) compared with men with neither osteopenia/osteoporosis nor sarcopenia.
Poggiogalle E et al. 2019 [29] USA	Cross-sectional study, Community-dwelling	Mean 92 ± 2 year *N* = 87 37(42.5)/50(57.5)	Muscle mass: DXA BMD: DXA	Sarcopenia: low ALM Osteopenia, osteoporosis: WHO criteria	31%	IGF-1	In osteosarcopenic men, IGF1-SDS values were lower than those in control males whereas IGF1-SDS were similar in the other body compositions phenotypes in female.
Balogun S. et al. 2019 [30] Australia	Prospective study, Community-dwelling	Mean 62.9 ± 7.4 *N* = 1032	Muscle mass: DXA Muscle strength: grip strength BMD: DXA	Sarcopenia: in the lowest 20% of the sex-specific distribution for ALM/BMI or grip strength Osteopenia, Osteoporosis: WHO criteria	8.3%	Mortality over 10 years Fractures over 10 years	Mortality risk was significantly higher only in participants with osteosarcopenia (RR = 1.49, 95% CI: 1.01–2.21) compared to without sarcopenia or osteopenia. Osteosarcopenia and osteodynapenia did not lead to a significantly greater fracture or mortality risk compared to having these conditions on their own.
Kobayashi K. et al. 2019 [31] Japan	Cross-sectional study, Community-dwelling	Mean 71.4 years *N* = 427 205(48.0)/222(52.0)	Muscle mass: DXA BMD: DXA	Sarcopenia: AWGS Osteoporosis: JOS-criteria	All subjects: 8% Females: 12% Males: 4%	Physical function	BMI and back muscle strength were significantly lower in osteosarcopenia than in sarcopenia alone (*p* < 0.05); and weight, BMI, body fat, grip strength, and back muscle strength were significantly lower in osteosarcopenia than in osteoporosis alone (*p* < 0.05).
Fahimfar N et al. et al. 2020 [32] Iran	Cross-sectional study, Community-dwelling	Osteosarcopenia: Mean 71.2 Non: Mean 68.3 *N* = 2353 1148(48.8)/1205(51.2)	Muscle mass: DXA Muscle strength: grip strength BMD: DXA	Sarcopenia: AWGS Osteopenia, osteoporosis: WHO criteria	Men: 33.8% Women: 33.9%	Risk factor of cardiovascular diseases	BMI (PR 0.84, 95% CI 0.81–0.88 in men and 0.77, 95% CI 0.74–0.80 in women), high-fat mass was positively associated with osteosarcopenia [PR 1.46 (95% CI 1.11–1.92) in men, and 2.25 (95% CI 1.71–2.95) in women], Physical activity in men (PR = 0.64, 95% CI 0.46, 0.88), diabetes in men (PR 1.33, 95% CI 1.04–1.69) was showed a direct association with osteosarcopenia.
Okamura H et al. 2020 [8] Japan	Cross-sectional study, Regularly visited University Hospital	Mean 77.07 *N* = 276	Muscle mass: DXA Muscle strength: grip strength BMD: DXA	Sarcopenia: AWGS Osteoporosis: JOS-criteria	19.6%	Frailty (Frailty Phenotype) Risk factors	Osteosarcopenia group had a greater risk of frailty than did those in the osteoporosis alone (OR 2.33; 95% CI, 1.13– 4.80, *p* = 0.028). Low BMI seemed to be the strongest factor related to the development of osteosarcopenia. Multiple logistic analyses revealed that patients aged 65–74 years who had comorbidities such as kidney dysfunction and high levels of HbA1c were at risk of developing osteosarcopenia.
Sepúlveda-Loyola W et al. 2020 [33] Australia	Cross-sectional study, Community-dwelling	Mean 77.9 ± 0.42 *N* = 253	Muscle mass: DXA Muscle strength: grip strength BMD: DXA	Sarcopenia: EWGSOP, EWGSOP2, FNIH Osteopenia, osteoporosis: WHO criteria	11–21%	Falls Past fractures in the past 5 years	Osteosarcopenia was associated with worse SPPB, TUG, FSS, limit of stability, and falls and fractures history. Osteosarcopenia (using the severe sarcopenia classification) conferred an increased rate of falls (OR from 2.83 to 3.63; *p* < 0.05 for all) and fractures (OR from 3.86 to 4.38; *p* < 0.05 for all) when employing the EWGSOP2 and FNIH definitions.
Salech F et al. 2020 [34] Chile	Observational study, Community-dwelling	Mean 72 ± 6.7 *N* = 1119	Muscle mass: DXA Muscle strength: grip strength BMD: DXA	Sarcopenia: EWGSOP Osteopenia, osteoporosis: WHO criteria	16.4%	Mortality Fracture Falls Functional limitations	Cox Regression analysis, the hazard ratio for death in people with osteosarcopenia was 2.48. Falls, fractures, and functional impairment were significantly more frequent in osteosarcopenic patients.

Abbreviations: BIA—Bioimpedance analysis; BMD—Bone mineral density; DXA—Dual-energy X-ray absorptiometry; AWGS—Asian Working Group for Sarcopenia; EWGSOP—European Working Group on Sarcopenia in Older People; WHO—World Health Organization; OR—Odds ratio; CI—Confidence interval; BMI—Body mass index; aLM—Appendicular lean mass; FNIH—Foundation for the National Institutes of Health; SPPB—Short Physical Performance Battery; GFST—Gerontopole Frailty Screening Tool; PTH—Parathyroid hormone; ASM—Appendicular skeletal mass; SD—Standard deviation; HR—hazard ratio; IGF1−SDS—Insulin-like growth factor 1 Standard Deviation Scores; RR—Relative risk; JOS—Japan Osteoporosis Society; TUG—Timed Up and Go test; FSS—Four-Square Step test.

**Table 2:** Assessment, prevalence, and impact of osteosarcopenia on clinical outcomes in a hospital setting.

Author, Year, Country	Design, Setting	Disease	Age Sample Size Male/Female, *N* (%)	Measurements	Diagnosis	Prevalence	Outcomes	Main Results
Yoo J Il et al. 2018 [35] Korea	Retrospective observational study, University hospital	Hip fracture	Mean 77.8 ± 9.7 *N* = 324 78(24.1)/246(75.9)	Muscle mass: BIA Muscle strength: grip strength BMD: DXA	Sarcopenia: AWGS Osteopenia, osteoporosis: WHO criteria	28.7%	1 year mortality	The 1-year mortality of osteosarcopenia (15.1%) was higher than that of other groups (normal: 7.8%, osteoporosis only: 5.1%, sarcopenia only: 10.3%).
Reiss J, et al. 2019 [40] Austria	Cross-sectional study, University hospital	Geriatric inpatients	Mean 80.6 ± 5.5 *N* = 141 84 (59.6)/57(40.4)	Muscle mass: DXA Muscle strength: grip strength BMD: DXA	Sarcopenia: EWGSOP Osteoporosis: WHO criteria	14.2%	Gait speed Hand grip strength Barthel index Body mass index Mini nutritional assessment-short form	The BMI and MNA-SF were lower in osteosarcopenia compared to sarcopenia or osteoporosis alone (*p* < 0.05) while there were no differences in functional criteria.
Saeki C. et al. 2019 [38] Japan	Cross-sectional study, General Hospital	Liver cirrhosis	Mean 70.5 (58.8–76.0) *N* = 142 90(63.4)/52(36.6)	Muscle mass: BIA Muscle strength: grip strength BMD: DXA	Sarcopenia: Japan Society of Hepatology criteria, AWGS, EWGSOP2 Osteopenia, osteoporosis: WHO criteria	21.8%	Vertebral fracture	Vertebral fracture occurred most frequently in patients with osteosarcopenia (61.3%) and least frequently in those without both sarcopenia and osteoporosis (15.8%).
Saeki C et al. 2020 [37] Japan	Cross-sectional study General Hospital	Chronic liver disease	Median 70 years *N* = 291 137(47.1)/154(52.9)	Muscle mass: BIA Muscle strength: grip strength BMD: DXA	Sarcopenia: Japan Society of Hepatology criteria Osteopenia, osteoporosis: WHO criteria	16.8%	Frailty	On multivariate analysis, frailty was an independent factor associated with osteosarcopenia (OR, 9.837; *p* < 0.001), and vice versa (OR, 10.069; *p* < 0.001).
Saeki C et al. 2020 [39] Japan	Cross-sectional study, University hospital and General Hospital	Primary biliary cholangitis	68.0 (56.5–73.0) *N* = 117 21(17.9)/96(82.1)	Muscle mass: DXA Muscle strength: grip strength BMD: DXA	Sarcopenia: the Japan Society of Hepatology guideline Osteoporosis: WHO criteria	15.4%	Vertebral fracture	Osteosarcopenia had significantly higher prevalence of vertebral fracture (55.6%) than those without both osteoporosis and sarcopenia (6.7%)
Di Monaco M et al. 2020 [36]	Cross-sectional study Research center	Hip fracture	79.7 ± 7.2 *N* = 350 (all women)	Muscle mass: DXA Muscle strength: grip strength BMD: DXA	Sarcopenia: FNIH Osteoporosis: T-score <−2.5SD	65.7%	Vertebral fracture (spine deformity index)	The presence of sarcopenia and osteoporosis was associated with a higher spine deformity index than the presence of only one of the 2 conditions

Abbreviations: BIA—Bioimpedance analysis; BMD—Bone mineral density; DXA—Dual-energy X-ray absorptiometry; AWGS—Asian Working Group for Sarcopenia; WHO—World Health Organization; EWGSOP—European Working Group on Sarcopenia in Older People; OR—Odds ratio; FNIH—Foundation for the National Institutes of Health; MNA-SF—Mini Nutritional Assessment-Short Form; SD—Standard deviation.

## Supplementary Materials

The following are available online at https://www.mdpi.com/2072-6643/13/2/291/s1, Table S1: Results of the quality assessment of study on osteosarcopenia and clinical outcomes. Table S2: Results of the quality assessment of study on osteosarcopenia and clinical outcomes in hospital setting.
