# Supplementary material for: Related Factors and Clinical Outcomes of Osteosarcopenia: A Narrative Review
Source: Nutrients. 2021 Jan 20;13(2):291. doi: 10.3390/nu13020291 (PMC7909576; doi:10.3390/nu13020291)
Supplement: Supplementary file 1 [file nutrients-13-00291-s001.pdf]

Supplementary Table S1. Results of the quality assessment of study on osteosarcopenia and clinical outcomes in community-dwelling older adults

Author	Criteria 1	Criteria 2	Criteria 3	Criteria 4	Criteria 5	Criteria 6	Criteria 7	Criteria 8	Criteria 9	Criteria 10	Criteria 11	Criteria 12	Criteria 13	Criteria 14	Quality rating
Wang, Y. J. et al. 2015	Y	Y	Y	Y	N	N	NA	NA	Y	NA	Y	NR	NR	N	Fair
Huo YR et al. 2015	Y	Y	NR	Y	N	N	NA	NA	Y	NA	Y	NR	NR	N	Fair
Drey M et al. 2016	Y	Y	NR	Y	N	N	NA	NA	Y	NA	Y	NR	NR	N	Fair
Szlejf C et al. 2017	Y	Y	Y	Y	N	N	NA	NA	Y	NA	Y	NR	N	N	Fair
Suriyaarachchi P et al. 2018	Y	Y	NR	Y	N	N	NA	NA	Y	NA	Y	NR	NR	N	Fair
Susan Park et al. 2018	Y	Y	N	Y	N	N	NA	NA	Y	NA	Y	NR	NR	N	Poor
Scott D et al. 2019	Y	Y	Y	Y	N	Y	Y	NA	Y	NA	Y	NR	Y	N	Good
Poggiogalle E et al. 2019	Y	Y	N	N	N	Y	NA	NA	Y	NA	Y	NR	NR	N	Fair
Balogun S. et al. 2019	Y	Y	Y	Y	N	Y	Y	NA	Y	NA	Y	NR	Y	N	Good
Kobayashi K. et al. 2019	Y	Y	NR	Y	N	N	NA	NA	Y	NA	Y	NR	NR	N	Fair

Fahimfar N et al. et al. 2020	Y	Y	Y	Y	N	NA	NA	NA	Y	NA	Y	NR	Y	N	Good
Okamura H et al. 2020	Y	Y	Y	Y	N	NA	NA	NA	Y	NA	Y	NR	NR	N	Fair
Sepúlveda-Loyola W et al. 2020	Y	Y	NR	Y	N	NA	NA	NA	Y	NA	Y	NR	Y	N	Fair
Salech F et al. 2020	Y	Y	Y	Y	N	NA	NA	NA	Y	NA	Y	NR	Y	N	Fair

Quality of the selected observational study was assessed using the National Institutes of Health (NIH) Quality Assessment tool for Observational Cohort and Cross-Sectional Studies (https://www.nhlbi.nih.gov/health-pro/guidelines/in-develop/cardiovascular-risk-reduction/tools/cohort). Criteria1. Was the research question or objective in this paper clearly stated? Criteria 2. Was the study population clearly specified and defined? Criteria 3. Was the participation rate of eligible persons at least 50%? Criteria 4. Were all the subjects selected or recruited from the same or similar populations (including the same time period)? Were inclusion and exclusion criteria for being in the study prespecified and applied uniformly to all participants? Criteria 5. Was a sample size justification, power description, or variance and effect estimates provided? Criteria 6. For the analyses in this paper, were the exposure(s) of interest measured prior to the outcome(s) being measured? Criteria 7. Was the timeframe sufficient so that one could reasonably expect to see an association between exposure and outcome if it existed? Criteria 8. For exposures that can vary in amount or level, did the study examine different levels of the exposure as related to the outcome (e.g., categories of exposure, or exposure measured as continuous variable)? Criteria 9. Were the exposure measures (independent variables) clearly defined, valid, reliable, and implemented consistently across all study participants? Criteria 10. Was the exposure(s) assessed more than once over time? Criteria 11. Were the outcome measures (dependent variables) clearly defined, valid, reliable, and implemented consistently across all study participants? Criteria 12. Were the outcome assessors blinded to the exposure status of participants? Criteria 13. Was loss to follow-up after baseline 20% or less? Criteria 14. Were key potential confounding variables measured and adjusted statistically for their impact on the relationship between exposure(s) and outcome(s)? The overall judgment is determined by Good, Fair, Poor. CD, cannot determine; NA, not applicable; NR, not reported.

Supplementary Table S2. Results of the quality assessment of study on osteosarcopenia and clinical outcomes in hospital setting

Author	Criteria 1	Criteria 2	Criteria 3	Criteria 4	Criteria 5	Criteria 6	Criteria 7	Criteria 8	Criteria 9	Criteria 10	Criteria 11	Criteria 12	Criteria 13	Criteria 14	Quality rating
Yoo J Il et al. 2018	Y	Y	Y	Y	N	Y	Y	NA	Y	NA	Y	NR	Y	N	Good
Reiss J, et al. 2019	Y	Y	Y	Y	N	NA	NA	NA	Y	NA	Y	NR	Y	N	Fair
Saeki C. et al. 2019	Y	Y	Y	Y	N	NA	NA	NA	Y	NA	Y	NR	Y	N	Good
Saeki C et al. 2020	Y	Y	Y	Y	N	NA	NA	NA	Y	NA	N	NR	Y	N	Fair
Saeki C et al. 2020	Y	Y	Y	Y	N	NA	NA	NA	Y	NA	N	NR	Y	N	Fair
Di Monaco M et al. 2020	Y	Y	Y	Y	N	NA	NA	NA	Y	NA	N	NR	Y	N	Fair

Quality of the selected observational study was assessed using the National Institutes of Health (NIH) Quality Assessment tool for Observational Cohort and Cross-Sectional Studies (https://www.nhlbi.nih.gov/health-pro/guidelines/in-develop/cardiovascular-risk-reduction/tools/cohort). Criteria1. Was the research question or objective in this paper clearly stated? Criteria 2. Was the study population clearly specified and defined? Criteria 3. Was the participation rate of eligible persons at least 50%? Criteria 4. Were all the subjects selected or recruited from the same or similar populations (including the same time period)? Were inclusion and exclusion criteria for being in the study prespecified and applied uniformly to all participants? Criteria 5. Was a sample size justification, power description, or variance and effect estimates provided? Criteria 6. For the analyses in this paper, were the exposure(s) of interest measured prior to the outcome(s) being measured? Criteria 7. Was the timeframe sufficient so that one could reasonably expect to see an association between exposure and outcome if it existed? Criteria 8. For exposures that can vary in amount or level, did the study examine different levels of the exposure as related to the outcome (e.g., categories of exposure, or exposure measured as continuous variable)? Criteria 9. Were the exposure measures (independent variables) clearly defined, valid, reliable, and implemented consistently across all study participants? Criteria 10. Was the exposure(s) assessed more than once over time? Criteria 11. Were the outcome measures (dependent variables) clearly defined, valid, reliable, and implemented consistently across all study

participants? Criteria 12. Were the outcome assessors blinded to the exposure status of participants? Criteria 13. Was loss to follow-up after baseline 20% or less? Criteria 14. Were key potential confounding variables measured and adjusted statistically for their impact on the relationship between exposure(s) and outcome(s)? The overall judgment is determined by Good, Fair, Poor. CD, cannot determine; NA, not applicable; NR, not reported.
